# Congenital Heart Disease and Impacts on Child Development

**DOI:** 10.5935/1678-9741.20160001

**Published:** 2016

**Authors:** Mariana Alievi Mari, Marcelo Matos Cascudo, João Carlos Alchieri

**Affiliations:** 1 Universidade Federal do Rio Grande do Norte (UFRN), Natal, RN, Brazil.; 2 Departamento de Cirurgia Cardiovascular do Instituto do Coração de Natal (INCOR/Natal), Natal, RN, Brazil.

**Keywords:** Child Development, Heart Defects, Congenital, Behavioral Medicine, Psychology, Child

## Abstract

**Objective:**

To evaluate the child development and evaluate a possible association with the commitment by biopsychosocial factors of children with and without congenital heart disease.

**Methods:**

Observational study of case-control with three groups: Group 1 - children with congenital heart disease without surgical correction; Group 2 - children with congenital heart disease who underwent surgery; and Group 3 - healthy children. Children were assessed by socio-demographic and clinical questionnaire and the Denver II Screening Test.

**Results:**

One hundred and twenty eight children were evaluated, 29 in Group 1, 43 in Group 2 and 56 in Group 3. Of the total, 51.56% are girls and ages ranged from two months to six years (median 24.5 months). Regarding the Denver II, the children with heart disease had more "suspicious" and "suspect/abnormal" ratings and in the group of healthy children 53.6% were considered with "normal" development (*P*≤0.0001). The biopsychosocial variables that were related to a possible developmental delay were gender (*P*=0.042), child's age (*P*=0.001) and income *per capita* (*P*=0.019).

**Conclusion:**

The results suggest that children with congenital heart disease are likely to have a developmental delay with significant difference between children who have undergone surgery and those awaiting surgery under clinical follow-up.

**Table:** 

**Abbreviations, acronyms & symbols**	
CHD	= Congenital heart disease
DDST	= Denver Developmental Screening Test
G1	= Group 1
G2	= Group 2
G3	= Group 3
SUS	= Brazilian National Health Care System

## INTRODUCTION

Child development is the result of the interaction of various aspects, including biological, psychological and social factors. The acquisition of new skills is related to the child's age and experienced interactions with other individuals of their social environment^[1]^. Studies have shown that assess environmental conditions and stimuli that are offered to children by their families can provide important data for the development of preventive and promotional health interventions^[2]^.

Considering the biological aspects, it can identify the many chronic diseases that affect the pediatric population, as in the case of congenital heart disease (CHD), for example. CHD covers a wide variety of anatomical and functional cardiac malformations. It is currently the most common in newborns alive, reaching 1% of the Brazilian population^[3,4]^. The presence of this disease is also linked to symptoms such as dyspnea, fatigue, dizziness, low weight, frequent respiratory infections, arrhythmia, and cyanosis that depending on the degree, can cause physical and motor inhibition constraints that directly affect the emotional and cognitive development^[5]^. In adittion to this, cultural factors can alter the development of the brain and should be considered as a dependent variable that influences and is influenced by environmental factors. Such factor is the socioeconomic level that includes education, nutritional status, quantity and stimulation of quality medical care, perinatal risks, occupation, family and social interaction and housing conditions styles^[2]^.

Children with CHD may show changes in their psychomotor development by pathophysiological factors such as low birth weight, cyanosis, among others, but also for chronic disease that impose numerous hospitalizations, repeated examinations, physical constraints and consequently, school and social withdrawal. Considering all this context, children with CHD, depending on the severity of the disease, can obtain significantly lower scores compared to children without disease with regard to changing child development^[6]^. This study aims to evaluate child development and verify a possible association with the commitment by biopsychosocial factors of children with and without CHD. Children were assessed by the Denver II Screening Test, published in 1992 and is configured as one of the most widely used instruments in the evaluation of children aged zero to six years old and is the result of an update and comprehensive review of the Denver Developmental Screening Test (DDST) published by Frankenburg et al.^[7]^ in 1967. The participants were children systematically treated by the Pediatric Service of INCOR/ Natal - Brasil and AMICO - Friends Association for Children's Heart Institutions that provide support for children with heart disease across the state of Rio Grande do Norte, in Brazil.

## METHODS

### Study design and participants

This research is an observational case-control study comparing the development of children with and without heart disease. They were included in Group 1 (G1) - children 0-6 years, of both genders, CHD, the waiting list for surgery, assisted by Brazilian National Health Care System (SUS); Group 2 (G2) - children aged 0-6 years old, of both genders, assisted by SUS, who have undergone at least one surgical procedure for CHD correction up to one year prior to application of the instruments of this study; and Group 3 (G3) - children 0-6 years, healthy, both genders, SUS users. The time between the surgery and the administration of the instruments was set from one month to one year after the completion of the first surgery because before one month the child is still in the immediate recovery of the procedure and after one year the evaluation of development of the benefits of the procedure can suffer interference from other variables. Children from G1 and G2 were recruited and selected in specialized services in cardiology Natal/RN and the children from G3 were recruited and selected in a Municipal Center for Child Education in the same city. Children with syndromes, with no neurological problems resulting from heart disease or children whose mothers or main caregivers was not present in the administration of the instruments were not included in the ratings in the three groups.

### Instruments

As instruments we used an biopsychosocial data questionnaire specifically developed for this research that includes social, demographic, psychological and clinical factors and the DENVER II Development Screening Test, which consists of 125 items, divided into four fields of functions: personal-social, fine-motor-adaptive, language and gross motor. Each of the 125 items is represented by a bar that contains the ages at which 25%, 50%, 75% and 90% of the children presented the suggested skills. To test administration, it was calculated the child's age in years and months and we drew a vertical line at the corresponding age. The amount of items varied according to the age of the child, and all administered items were cut by the vertical line and at least three items completely left of the line. Published in 1992, DENVER II is currently one of the instruments used in the evaluation of children aged zero to six years old and is the result of an update and comprehensive review of the DDST published by Frankenburg et al.^[7]^.

### Statistical analysis

In order to analyze the data and sample characterization, descriptive statistics and central tendency (frequency and percentage, median and percentiles, mean and standard deviation) were used.

In order to verify the mean differences between groups, the study sample did not show a normal distribution, non-parametric tests were performed. Pearson's Chi-square was employed for independent and categorical samples and the Kruskal-Wallis and Mann-Whitney Test to compare continuous variables. The paternal participation was assessed by the Shapiro-Wilk Normality Test to determine if this set of data were a normal distribution and after the Chi-square analysis for comparison between groups. The one-way Analysis of Variance ANOVA was performed to compare the average age of parents between groups.

The associations between biopsychosocial variables and the Denver II were analyzed by the Fisher's Exact Test considering the sample size. This statistical analysis excluded 17 participants who received review "impossible to test" in the Denver II, considering that this variable could be a confounding bias.

### Ethical aspects

The study was approved by the Ethics Research Committee of the Onofre Lopes Hospital/Natal-RN (CAAE No. 01120112.1.0000.5292) in July 2013. Before the administration of the instruments, the mother or the person responsible for the child was instructed about the research procedures and invited to sign the Informed Consent.

## RESULTS

The results of the 128 children were collected, 29 (22.66%) belonging to the G1 (preoperative children with heart disease), 43 (33.59%) from G2 (postoperative children with heart disease) and 56 (43.75%) belonging to the G3 (healthy children). Of the total, 66 (51.56%) were female, and the ages ranged from two months to six years (median 24.5 months). The characterization of the three groups is shown in Table 1.

**Table 1:** Sample characterization (n = 128).

	G1 (n=29)	G2 (n=43)	G3 (n=56)	*P*
Female gender	20 (69)	17 (39.5)	29 (51.8)	0.047
Age				
1 to 11 months	9 (31)	18 (41.9)	5 (8.9)	
12 to 23 months	6 (20.7)	12 (27.8)	11 (19.6)	<0.0001
24 to 47 months	12 (41.4)	10 (23.3)	18 (32.2)	
48 to 72 months	2 (6.9)	3 (7)	22 (39.3)	
Mother's age	26.34 (8.13)^(a)^	29 (6.88)^(a)^	29.57 (8)^(a)^	0.177
Father's age	28.96 (7.69)^(a)^	31.73 (7.64)^(a)^	32.1 (9.82)^(a)^	0.280
Mother's Education (years)	9 (6-11)^(b)^	10 (8-11)^(b)^	10 (7-11.75)^(b)^	0.295
Father's Education (years)	9.5 (5-11)^(b)^	8 (5-11)^(b)^	9 (6-11)^(b)^	0.427
Number of Children	2 (1-3)^(b)^	2 (1-3)^(b)^	2 (1-3)^(b)^	0.537
Only child	13 (44.8)	14 (32.6)	19 (33.9)	
The youngest child	14 (48.3)	24 (55.8)	24 (42.9)	0.413
*Per capita* income	R$179.00	R$200.00	R$289.88	0.042
	(141.42-242.00)^(b)^	(103.71-289.71)^(b)^	(136.25-440.31)^(b)^	
Unplanned pregnancy	15 (51.7)	24 (55.6)	40 (71.4)	0.126
Prenatal	28 (96.6)	43 (100)	50 (89.3)	0.024
No Problems during pregnancy	15 (51.7)	25 (58.1)	43 (76.8)	0.036
No Complications at birth	18 (62.1)	32 (74.4)	49 (87.5)	0.025
No history of Use of Substance during pregnancy	24 (82.8)	41 (95.3)	47 (83.9)	0.120

(a) mean and standard deviation

(b) median and percentiles

Table 2 presents an overview of the children with heart disease who composed the G1 and G2.

**Table 2:** Overview of congenital heart disease (G1 and G2) (n=72).

	G1 %	G2 %	*P*
Classification of diseases			
Cyanotic	44.8	41.9	0.496
Acyanotic	55.2	58.1	
Heart disease discovery			
1^st^ month of life	58.7	46.5	
2 months to 1 year	31	37.2	0.169
After 1 year of life	10.3	7	
During gestation	-	9.3	
Number of admissions	1 (1-1)^(a)^	2 (1-3)^(a)^	<0.0001
Information received			
Yes	62.1	83.7	0.036
Understands the disease			
Yes	27.6	37.2	0.405
Partially	48.3	32.6	
Behavioral change			
Yes	21.4	85.7	<0.0001
Type of changes			
Positive	7.1	50	
Negative	14.3	35.7	<0.0001
Limits the child's activities			
Yes	63	61.9	0.568

(a) median and percentiles

In G3, 15 (26.5%) of the children have undergone hospitalization, 8 of them (14.4%) were due to respiratory problems. The number of admissions ranged from none to five hospitalizations, of which, 10.6% had a hospitalization and 7.2% had two or more hospitalizations.

A development review by Denver II Screening Test of the three groups is shown in Table 3.

**Table 3:** General Result of the Denver II Assessment (n = 128).

	G1	G2	G3	*P*
Normal	7 (24.1)	9 (20.8)	30 (53.6)	
Suspect	11 (38)	10 (23.3)	15 (26.8)	<0.0001
Abnormal	8 (27.6)	18 (41.9)	3 (5.3)	
Impossible Test	3 (10.3)	6 (14)	8 (14.3)	

By linking the biopsychosocial variables with Denver II assessments, the variables gender, age and income *per capita*, shown in Table 4, presented significant difference (*P*=0.042, *P*=0.001, *P*=0.019). Inferential statistics were performed with 111 participants, since 17 children were characterized as "impossible to test", thus they were excluded for not being a confounding bias.

**Table 4:** Gender and age related to Denver II (n = 111).

	Normal n (%)	Suspect n (%)	Abnormal n (%)	*P*
Gender				
Female	30 (52.6)	14 (24.6)	13 (22.8)	0.042
Men	16 (29.6)	22 (40.7)	16 (29.6)	
Age				
1 to 11 months	10 (31.2)	8 (25)	14 (43.8)	
12 to 23 months	9 (36)	6 (24)	10 (40)	0.001
24 to 47 months	13 (46.4)	10 (35.7)	5 (17.9)	
48 to 72 months	14 (53.8)	12 (46.2)	-	

**Table 5:** Relation between income *per capita* and income for the Denver II * (n = 111).

	Normal	Suspect	Abnormal
Median	R$ 287.71	R$ 216.66	R$ 181.50
Percentiles	R$171.30/R$ 434,86	R$ 100.00/R$ 286.37	R$ 133.92/R$ 242.00
Minimum	R$ 0.00	R$ 22.40	R$ 36.00
Maximum	R$ 1000.00	R$ 907.00	R$ 500.00

* P=0.019

Age and education of the parents showed no significant difference in relation to Denver II classification. The variables that did not show statistically significant differences were: variables related to prenatal and postpartum (planned pregnancy had problems in pregnancy, mode of delivery, premature baby). Complications after birth showed a trend towards significance (*P*=0.065), and 44% of children who had complications were classified as "suspect/abnormal".

In the analyzes carried out in the G1 and G2 there were no significant differences in the psychosocial variables, age at diagnosis, parents have received information about the disease, understanding disease and the child's treatment, limit activities and change of behavior after diagnosis.

In G3 50% of children who were hospitalized at least once had their Denver II characterized as "suspect" (*P*=0.025). It was found that the more the child spent admitted to the hospital, higher the frequency of "suspicious" and "suspect/abnormal" ratings (*P*=0.023).

## DISCUSSION

### Sampling feature

The *per capita* income have presented statistical difference among the three groups which may be related to the mothers of children with chronic disease that mostly do not have a job to devote themselves to child care. The family member when become the caretaker of a child with chronic illness has their life affected in many ways, as interference in work and personal life. In most cases, the family member who takes care is the mother and, in that sense, the employment disruption is not unusual which entails a change in family economic organization^[8]^.

The fact that the G1 and G2 babies have more complications at birth is due to cardiovascular disorders such as dyspnea, cyanosis, irregular heartbeats that are one of the main symptoms that appear soon after birth signaling changes^[3,9]^.

In G1 and G2, the most common heart disease were: tetralogy of Fallot, patent ductus arteriosus, ventricular septal defect, atrial septal defect and atrial septal defect associated with pulmonary stenosis. These results are consistent with findings in the literature that state that these diseases are among the most common ones^[3,10]^.

Unlike other studies that classify the groups of children with heart disease by disease severity or hemodynamic compromise, this study proposed a separate action related to the surgical procedure, regardless of diagnosis. In a study to assess the psychomotor development after repair or palliative procedure with 243 five-year-old children with heart disease, patients were divided into two groups: those with biventricular repair and single ventricle repair^[11]^. In another study, the patients were divided by the authors into two groups: the ones with hemodynamic consequences and without hemodynamic effect^[12]^. As the present study aimed to verify psychosocial factors linked to illness and not biological or physiological factors of heart disease classification, we divided the children into two categories: children before and after heart surgeries.

Regarding the age, most of the patients had their disease discovered in the first month of life until the child is one year old. However, it is noteworthy that only in G2 (postoperative children) we found mothers who discovered the disease during pregnancy (9.3%). This may be related to the fact that the intrauterine diagnosis is more related to serious heart disease and need immediate surgical intervention, as in the case of hypoplastic left heart that the rough decreased left ventricular cavity allows easy viewing even for professional who is not an expert in the field^[13]^.

The mothers of children who had undergone surgery indicated that they had received more information than those who were on the list of preoperative patients. In a literature review study on the need for information and parents to support children with CHD, the authors, after analysis of items, consider that the parents' knowledge is incomplete and that this knowledge is affected by the severity of heart disease. It is important to note that knowledge is a broad field that involves knowing from information on diagnosis to the clinical consequences^[14]^.

Information from health professionals are usually restricted to the physical aspects of treatment such as food, observation of signs related to pathology and notions of hygiene to prevent infection. These guidelines leave mothers/caregivers restricted to physical symptoms increasing attention and care, imposing a number of limitations, overprotecting the child^[15]^. Lack of adequate or appropriate information can be related to the large number of mothers that limit the child's activities, and in this study most mothers of G1 and G2 does not let the child cry, crawl, walk or run, as well as to changes in behavior after diagnosis (87% in G2). Although the majority of mothers have shown positive changes, a significant number reported negative changes.

### Developmental profile

The Denver II tracks possible developmental delays. Considering that the child development is a broad and complex process, the developmental delay should be confirmed, through instruments that can provide a proper study, ensuring the diagnosis^[7]^. The choice of this instrument was made from the realization that there are no available tools for psychologists to assess the development of children under 5 years of age. As long as the population of CHD suffers from the interference of the disease from birth, it is advisable for development studies to assess these children before the first year of life^[16,17]^.

Based on the results, we can say that children with CHD have probable developmental delay, with a significant difference between children who underwent surgery for correction of cardiac malformation and those awaiting surgery, under clinical follow-up. The results obtained are compatible with other studies, though the division criterion of the groups was different regarding the researches. In a study on the development of children with CHD held in New York, 64 children were assessed using the Denver II, divided into: CHD which required surgical or catheter intervention and CHD without hemodynamic repercussions. As a result it was observed that 54% of the most serious children were classified as "abnormal, doubtful or untestable" and in the group of children without hemodynamic repercussion, 86% were characterized as "normal". It was concluded that children with complex CHD are more likely to delay risk than children with CHD who are not hemodynamically impaired^[12]^.

Similar work aimed to evaluate physical and neurological growth parameters in infants and children with CHD and the effect of hemodynamic status on these aspects through the Denver II. By comparing healthy children to those with heart disease, the authors found that children with cardiac problems hemodynamically impaired had more "abnormal" ratings than those in the group without hemodynamic impairment (*P*≤0.0001) and that the latter group resembled the results with the control group^[18]^. A longitudinal study using the Denver II evaluated 20 infants with CHD at three different times: 24 hours prior to heart surgery, the intensive care unit discharge and 3-6 months after surgery. Of the 20 infants evaluated, 15 had altered neurological examination and developmental delay before surgery, whose normalization was observed only six months after the procedure in six participants. The authors concluded that after five months on average, the frequency of surgery was reduced among children with Denver II "suspect" delay from 75% to 55%^[19]^.

This study points to a possible interference resulting from surgical procedures and convalescence in child development, regardless of the hemodynamic status or disease severity. Regarding the possible consequences of hospitalization and invasive procedures (catheterization, anesthesia and surgery) may subject the patient and family to a series of disturbing situations added to the previously adverse experiences, can cause great damage^[3]^.

### Biopsychosocial variables and child development

When comparing biopsychosocial variables to the results of the Denver II, we could find some aspects that are significantly linked to possible delay in development and other evidence in this study showed no statistical association.

Two factors that were associated with developmental delay were gender (*P*=0.042) and age (*P*=0.001). It is observed that most boys focused on the classification of "suspicious" and "suspect/ abnormal". Regarding gender, studies show that girls tend to do better in school than boys and that the differences become more apparent after the age of three, but on average, boys and girls are more alike than different^[1]^. In a survey that evaluated children in Feira de Santana (Bahia, Brazil) by the Denver II, an association between male and a worse performance in the test was found^[20]^.

The difference between the ages can be seen from some concepts related to childhood development. Biological factors predominate over social ones early in life. As soon as the interactions are established, primarily through language, this becomes a major role in the development of a child. Another aspect is that the relationship and interaction with adults make the child develop cognitive skills through shared experiences^[1]^. Relying on these theories is possible to think that as the child grows, they will develop skills and competencies that will interfere with their performance.

Family income is configured as an important determining factor in child development. Evidently, the lower per capita family income is related to higher frequency of developmental delay. This finding seems to be almost a consensus among scholars theme^[2,20-22]^. The socioeconomic status affects the processes and outcomes of development indirectly through the types of homes and neighborhoods in which people live and the quality of nutrition, medical care and schooling available^[1]^.

Aspects related to age and educational level of the parents, number of children per family and care before and after delivery were not significantly associated with the results of the development test. Other variables that did not show a significant relationship with developmental delays were: breastfeeding, mother's health problems and use of controlled medication or use of substances such as alcohol, tobacco and drugs during pregnancy. These factors may not have been shown to be associated with changes in development for mitigating characteristics of the environment, aspects of development that contained the difficulties, and also the population. Thus, it can be considered that in this population the most important risk factor was the presence of the disease, since the three groups were homogenous regarding the variables mentioned above.

However, one factor that showed a tendency to be related to child development was the baby having experienced complications after birth (*P*=0.065). It is believed that this issue is more associated with children with CHD, which disease characteristics may be showing cardiorespiratory changes within the first hours of life^[3,9]^.

When dealing with variables that only made up the group of children with heart disease (G1 and G2), different from what was imagined in the early phase of the study, aspects such as age at diagnosis, type of heart disease, having undergone hemodynamic procedures or not, information to parents about heart disease, understanding the disease and treatment, limitation of activities for children and the child with changes in behavior were not associated with changes in development. The fact that these variables do not affect the development of children with CHD may be related to protective factors and resilience.

Protective factors may be linked to individual features that reduce the effect of risk, since resilience is related to individual protective factors that predict positive consequences in individuals exposed to a risk context^[23]^. In a study of chronic adult patients where the resilience was measured through a scale, patients show high levels of resiliency and personal abilities and skills to handle the health status^[24]^. Although it is very difficult to measure resilience in children, it is possible to say that the initial experience of a child is important, but children can be remarkably resilient^[1]^. The own evolutionary/sociobiological theory states that humans possess adaptive mechanisms to survive and this may be related to protective factors^[1]^.

However, the survey shows that healthy children (G3) who were hospitalized for health care tend to exhibit behaviors suggestive of a developmental delay and more, the greater the number of hospitalizations that children faced, the they were characterized as "suspect" and "suspect/abnormal". Hospitalization in children is a matter studied since the 60's and 70's^[9]^. The immature thought process of a child leads them to understand the staff, equipment and procedures they are submitted during the hospitalization period in a wrong way. The different reactions, depending on the age, will be influenced by the personal characteristics of the child's relationship with parents and the approach to hospital situation^[9]^. In a study that assessed the impact of hospitalization in children 1-5 years old, it was found that both in the group of children with or without someone accompanying them, the most observed behaviors were crying, loss of appetite, rapid heartbeat, vomiting, insomnia and hyperthermia^[25]^. What in fact is represented here is that a process of illness and hospitalization has strong influences on the daily life of a child, and may cause damage depending on how these issues have been experienced.

## CONCLUSION

It was found that surgery is a traumatic event and brings changes in the routines of children and their families, with significantly influence on their psychomotor development. The results showed that despite the development being influenced by biological conditions of disease and treatment, children with heart disease studied showed that social and psychological factors were not able to interfere significantly in the development. This question suggests that children with heart disease, although it is a vulnerable group from the biological point of view, may prove resilient to those who experienced disorders. Family support is important, but the parents need to receive anticipated guidance on know how to deal appropriately with these situations, in order to deal with their child's growth and development, as well as to improve their care and to detect problems early.

As possible limitations identifies the sample size resulting from logistics, operating characteristics of the sampling sites in addition to the development assessment process. Characterize with certainty the assessment of the development in a single moment could compromise the understanding of behavioral expressions.

Based on the presented issues, identifies the need for further investigations as early assessment, systematic, longitudinal development of the fields with a view to evidence appropriate initial support and psychological preparation of the nuclear family and the child. It is important to have the realization of development monitoring studies of children with heart disease in other realities, such as patients with more differentiated, assisted by health plans and other states, characterizing the expression or absence of intervening variables.

The development process is broad and complex and investigate these issues in a risk population such as patients with CHD involves a number of variables that through further research need to be developed to provide advance in the understanding of this issue.

**Table:** 

**Authors' roles & responsibilities**	
MAM	Study design; implementation of projects and/or experiments; Analysis and/or interpretation of data; manuscript writing or critical review of its contents; final approval of the manuscript
MMC	Final approval of the manuscript
JCA	Manuscript writing or critical review of its contents; final approval of the manuscript
